# Centering health equity in large language model deployment

**DOI:** 10.1371/journal.pdig.0000367

**Published:** 2023-10-24

**Authors:** Nina Singh, Katharine Lawrence, Safiya Richardson, Devin M. Mann

**Affiliations:** 1 Department of Population Health, New York University Grossman School of Medicine, New York, New York, United States of America; 2 Medical Center Information Technology, New York University Langone Health, New York, New York, United States of America; Harvard University, UNITED STATES

Early buzz around ChatGPT’s [1] proficiency at healthcare tasks [2–4] has turned into rapid deployment of large language model (LLM) technology across healthcare. Multiple healthcare systems including UC San Diego Health, Stanford Health Care, and University of Wisconsin Health are already piloting the use of GPT-4 to draft responses to patient messages [5]. Others, including The University of Kansas Health System, are piloting similar LLMs to support clinical documentation [6].

Early conversations about LLMs in healthcare tended towards extremes, either lauding their breathtaking potential or lamenting the threat they pose to human-centric care. More recently, nuanced narratives regarding the ethical implications of LLMs have started to emerge [7,8]. Still missing from the conversation, though, is a grounded discussion of the pragmatic equity implications of LLM deployment in real-world contexts. In this perspective, we explore various health equity risks generated by the practical use of LLMs in healthcare and offer risk mitigation recommendations. We define health equity using Dr. Braveman’s definition: the “absence of differences in health between the most advantaged group in a given category and all others” [9].

Equity risks originating in the *development* of LLMs are widely discussed, with substantial dialogue dedicated to intrinsic equity and general ethical considerations of how, why, and with what data and algorithmic design choices these models should be developed [10,11]. Equity risks in AI model *deployment* are less commonly discussed. These emergent risks arise from pragmatic use by end users (patients, clinicians, and healthcare systems) in real-world settings. It is critical to understand emergent equity risks of LLMs to avoid replicating inequities that resulted from the use of other technologies in medicine [12].

For patients, LLMs have a remarkable ability to generate human-like responses and summarize complex or hard-to-find information [2,4], providing automated care with potential to alleviate disparities in access, understandability, and personalization. However, if minoritized populations who have experienced barriers to accessing traditional care are disproportionately more likely to seek care from a free chatbot rather than a licensed provider this could result in a uniquely biased care experience–with higher data privacy risks [13], biased outputs, misinformation, or personally offensive content from models trained on biased data [14–16]. This could perpetuate negative care experiences, medical disenfranchisement and distrust.

For clinicians, LLM-supported tools offer efficiency gains that may help address issues of burnout and dissatisfaction. For example, DocsGPT [17] automates high-burden, low-cognition tasks such as composing prior authorization letters. However, the choice to use LLMs versus not based on patient language, health literacy, or insurance coverage could widen existing disparities. Additionally, misguided clinician use of public LLMs could provide models with patient information that can become vulnerable to extraction [13].

For healthcare systems, LLM-supported technologies offer large-scale automation of patient communication, increasing customer relationship management efficiency and potentially improving value-based care metrics; these tools could also enable novel strategies to address health disparities, such as proactively connecting patients with identified social vulnerabilities with relevant resources. Unfortunately, competing priorities in care delivery for healthcare systems (eg revenue generation) too often result in the deprioritization of health equity efforts, including in the use of novel technologies. For example, as healthcare systems experiment with charging patients for messaging providers, there is a risk of minoritized patients preferentially turning to search engines and chatbots for medical advice. Healthcare systems that adopt widespread direct-to-patient LLM strategies that remove clinicians entirely from patient exchanges also risk impersonal, inaccurate, or biased [14–16] communications that would further ostracize minoritized patients from their providers and the experience of care delivery in general.

Finally, LLM products that organizations purchase for clinicians will contain behind-the-scenes prompting that shape how equitable responses are. While end users of AI models have traditionally had little power to alter the outputs of the models they’ve used, prompting has changed that dynamic [18]. Prompting enhances the performance of general-purpose models on healthcare tasks [18–20] and can change model accuracy, safety, and bias [18]. LLM vendors can thus be split into two categories: 1) LLM creators (who make general purpose LLMs, such as GPT-4) and 2) LLM product developers, who build upon existing LLMs to optimize prompts for clinical utility. There is a serious risk of inequitable prompts being created and used at mass scale as LLM product developers develop and sell their models to healthcare organizations. For example, a recent study found that when prompted with symptoms of high-risk chest pain and a patient’s insurance status, Chat-GPT correctly triaged insured patients to the emergency department but inappropriately suggested that uninsured patients either present to a community health center (less costly treatment venue) or the emergency department [21].

Ultimately, emergent equity risks in LLM deployment are complex and may be difficult to anticipate as novel use cases arise. Organizations such as the White House and National Academy of Medicine are working toward comprehensive frameworks for the ethical use of AI. However, in the meantime healthcare systems have a responsibility to engage in iterative risk mitigation around health equity as they rapidly deploy LLMs. Recommendations for addressing emergent equity issues in LLM deployment include:

## 1) Thoroughly evaluate LLM products and vendors for equity-related risks

Evaluating vendors can be facilitated by equity-informed requests for proposals or other intake tools designed to probe for equity-related concerns in product development, key performance indicators, and internal values. For LLM-supported products, this would include questions regarding the source and diversity of the training data (e.g. [22]), process for bias testing in technical development, post-deployment bias mitigation practice, and company policies regarding data transparency and explainability. Cedars-Sinai [23] and other hospitals are developing internal committees to review the ethics implications of all new artificial intelligence products before going live with them. Making sure that these committees have training in equity specifically will be important going forward.

## 2) Monitor LLMs rigorously during deployment

When using LLMs in practice, healthcare systems should establish robust post-deployment monitoring protocols for disparities in access, utilization, or outcomes. Additionally, they should work with vendors to regularly monitor model responses, both by humans trained to systematically evaluate for bias and by models trained to detect inequitable responses (e.g. responses that more often tell women that their reported symptoms are likely psychogenic than men). Patients and clinicians should retain the option to report biased responses at any time. Epic has created a forum for healthcare systems to share the GPT prompts that they are using with each other [24]. This forum and other similar ones could be expanded so that healthcare systems could share experiences with deploying different prompts, including any biases found, and collaboratively move toward more equitable prompts. Further research on more specific equity metrics (given the essentially infinite output space of LLMs) is warranted, and healthcare systems should advocate for funding for this work. In combination, these equity metrics should inform continuous improvement of the model and result in model termination if mitigation efforts are not possible.

## 3) Prepare clinicians to mitigate bias

While some healthcare systems may rely entirely on out-of-the-box prompt libraries (e.g. DocsGPT [17]), most will likely deploy both out-of-the-box and custom prompts. This relationship offers unique opportunities for clinician-users to identify and mitigate LLM bias. Beth Israel Deaconess Medical Center is starting to intentionally engage medical trainees with LLMs to allow them to better understand what they can and cannot do [25], and NYU Langone Health has educated various types of staff members throughout the healthcare system and partnered with them in developing and testing their own LLM ideas responsibly within a private and secure GPT instance [26]. Universal training in AI implicit bias as healthcare systems start to engage clinicians in LLM use can help ensure understanding of inherent biases in LLM tools and equip clinicians with mitigation strategies. A group of clinician champions should be specifically trained on prompt engineering, to support more targeted bias mitigation. Just as physician builders were trained on configuring certain aspects of electronic health records (dashboards, smartphrases, etc.), select clinicians should be trained to steward effective and equitable prompts for their healthcare systems. At a national level, professional societies can also be engaged to identify what data are necessary to create the most effective and equitable prompts for each of their specialty’s most common use cases.

LLMs are being rapidly operationalized, with tremendous potential to improve healthcare access, satisfaction, and outcomes. However, they also have the potential to amplify inequities by promoting digital health segregation and reinforcing underlying biases in the digital world. We must break the cycle of addressing health equity issues only after harm has been done and deliberately center equity in large language model deployment before it is too late.
